# Hierarchical integrated and segregated processing in the functional brain default mode network within attention-deficit/hyperactivity disorder

**DOI:** 10.1371/journal.pone.0222414

**Published:** 2019-09-12

**Authors:** Yongchen Fan, Rong Wang, Pan Lin, Ying Wu

**Affiliations:** 1 State Key Laboratory for Strength and Vibration of Mechanical Structures, Xi’an Jiaotong University, Xi’an, China; 2 School of Aerospace Engineering, Xi’an Jiaotong University, Xi’an, China; 3 College of Science, Xi’an University of Science and Technology, Xi’an, China; 4 Department of Psychology and Cognition and Human Behavior Key Laboratory of Hunan Province, Hunan Normal University, Hunan, China; 5 National Demonstration Center for Experimental Mechanics Education, Xi’an Jiaotong University, Xi’an, China; University of Cambridge, UNITED KINGDOM

## Abstract

The hierarchical modular organization of functional networks in the brain is crucial for supporting diverse cognitive functions. Functional disorders in the brain are associated with an abnormal hierarchical modular organization. The default mode network (DMN) is a complex dynamic network that is linked to specialized cognitive functions and clinically relevant information. In this study, we hypothesize that hierarchical functional segregation and integration of the DMN within attention-deficit/hyperactivity disorder (ADHD) is abnormal. We investigated topological metrics of both segregation and integration in different hierarchical subnetworks of the DMN between patients with ADHD and healthy controls. We found that the hierarchical functional integration and segregation of the DMN decreased and increased, respectively, in ADHD. Our results also indicated that the abnormalities in the DMN are intrinsically caused by changes in functional segregation and integration in its higher-level subnetworks. To better understand the temporally dynamic changes in the hierarchical functional integration and segregation of the DMN within ADHD, we further analyzed the dynamic transitions between functional segregation and integration. We found that the adaptive reorganizational ability of brain network states decreased in ADHD patients, which indicated less adaptive regulation between the DMN subnetworks in ADHD for supporting correspondingly normal cognitive function. From the perspective of hierarchical functional segregation and integration, our results further provide evidence to support dysfunctional brain cognitive functions within ADHD linked to brain network segregation and integration.

## Introduction

Attention-deficit/hyperactivity disorder (ADHD) is one of the most common and severe impairments of psychological development in children, with a prevalence of 0.1–8.1% worldwide; further, approximately 50% of patients are affected until adulthood [1]. Although there are argues about whether ADHD is a mental disorder or an extreme expression of normal human behavior, there is no doubt that ADHD patients have serious impairments not only in health but also in education and other aspects of life, manifesting as impaired attention and overactivity symptoms [2]. However, the etiology of ADHD is thus far unclear and requires further study.

Many studies have shown that ADHD is caused by abnormal communication within functional networks of the brain [3, 4]. Brain network reorganization shaped by mental disorders causes abnormal brain network segregation and integration [5]. Brain network segregation is the processing of multimodal information within local network communities. In addition, the brain promotes functional integration by enabling global communication between these communities [6, 7]. The balance of brain network segregation and integration plays an important role in supporting brain cognitive functions [8, 9]. Previous studies have demonstrated that ADHD patients exhibit decreased functional integration and increased segregation of brain networks compared to healthy subjects [10–13]. Our previous study also indicated the dysfunction of brain network communities in ADHD [4].

Recently, several studies have suggested that brain networks possess a hierarchical modular organization [14, 15], which allows brain networks to be represented as an economical wiring diagram and has several general advantages, including greater robustness, adaptivity, and evolvability of network functions [16]. Characterizing the hierarchical modular organization of brain networks is important for gaining a better understanding of brain network segregation and integration [7, 17]. However, to date, few studies have examined dysfunction of hierarchical functional segregation and integration within ADHD.

Previous studies have suggested that an abnormal default mode network (DMN) plays a critical role in mental disorders, such as depression and ADHD [3, 18–20]. The DMN contains several specific brain regions, consisting of parts of the posterior cingulate cortex, the precuneus, the ventromedial prefrontal cortex, the left and right inferior parietal lobes and the hippocampal areas. These brain regions are normally activated during the resting state and deactivated during task states [21–25]. Resting state functional magnetic resonance imaging (rs-fMRI) studies have suggested that adults with ADHD exhibit reduced coherence and abnormal connectivity between brain regions of the DMN [26–29]. The abnormal mechanisms underlying the functional networks in ADHD remains unclear from the perspective of functional separation and integration in hierarchical organization of the DMN.

In this study, we hypothesize that the hierarchical functional segregation and integration of the DMN within ADHD is abnormal. We investigate the difference in segregation and integration between patients with ADHD and healthy controls by analyzing topological metrics in the hierarchical subnetworks of the DMN topological metrics. In addition, we compare the dynamic transitions between segregated and integrated processing in the hierarchical DMN in ADHD and control subjects.

## Materials and methods

### fMRI acquisition

The resting-state fMRI data used in this research were released to the open-access “1000 Functional Connectomes Project” (http://fcon_1000.projects.nitrc.org/indi/CoRR/html/ipcas_1.html.) by Milham and Castellanos in December, 2009. These data were acquired from 24 ADHD patients and 24 healthy subjects (control subjects) in the resting state by a 3T Siemens scanner. The ADHD patients in this dataset were evaluated with the Clinical Interview for DSM-IV (SCID), the Symptom Checklist-90-Revised (SCL-90-R) and the Adult ADHD Clinical Diagnostic Scale (ACDS). It should be noted that we used pwr.t.test, a function in R statistical software, to calculate the power in the present analysis with the following parameters: number of samples in each group = 24, large effect size = 0.8, significance level = 0.05, power of test = 0.86. In general, the sample size is acceptable if the testing power calculated is more than 0.8. Image scans contained 39 slices and 192 time points with TR = 2 s, TE = 25 ms, flip angle = 90°, matrix = 64 × 64, FOV = 192 mm^2^, voxel size = 3 × 3 × 3 mm^3^, and a final time of 390 s.

### fMRI data preprocessing

The fMRI data were preprocessed using AFNI (http://afni.nimh.nih.gov/afni/) and FSL (http://www.fmrib.ox.ac.uk/fsl/) according to standard preprocessing protocols. First, the first four volumes were removed from the analysis to ensure the initial stabilization of the fMRI signal. Then, through a 3D image realignment with the AFNI program 3dvolreg function, motion correction was carried out for each subject. Images acquired from echo-planar imaging (EPI) were motion and slice-timing corrected, and spatially smoothed using a Gaussian kernel of 6 mm full width at half maximum (FWHM). To minimize the effects of low-frequency drift and high-frequency physiological noise, temporal bandpass filtering (0.01 Hz < f < 0.08 Hz) was performed. As the global component of the fMRI fluctuations measured during the resting state is tightly coupled with the underlying neural activity [30], it remains controversial to use global signal regression as a preprocessing step in rs-fMRI analyses. Thus, the whole-brain global signal was used in this analysis.

### Construction of the brain functional network

The brain was partitioned into 90 regions of interest (ROIs) with the AAL (Automated Anatomical Labeling) atlas [31], resulting in the DMN system containing 28 ROIs. The rs-fMRI time series for each ROI was obtained by averaging the voxel time series within each ROI, and the functional connectivity between ROIs was constructed by calculating the Pearson correlation coefficient:

$$\rho_{X,Y}=\frac{\sum_{t=1}^{N}\left(X(t)-\bar{X})(Y(t)-\bar{Y}\right)}{\sqrt{\sum_{t=1}^{N}{\left(X(t)-\bar{X}\right)}^{2}\sum_{t=1}^{N}{\left(Y(t)-\bar{Y}\right)}^{2}}}$$ (1)

where *t* is the time point, *N* stands for the total number of time points, *X* and *Y* are the rs-fMRI time series for different ROIs, and $\bar{X}$ and $\bar{Y}$ are the average values corresponding to these time series. Here, nearly all correlation values are positive due to not performing a regression on the whole-brain global signal.

### Hierarchical subnetworks of the DMN

**Fig 1:**
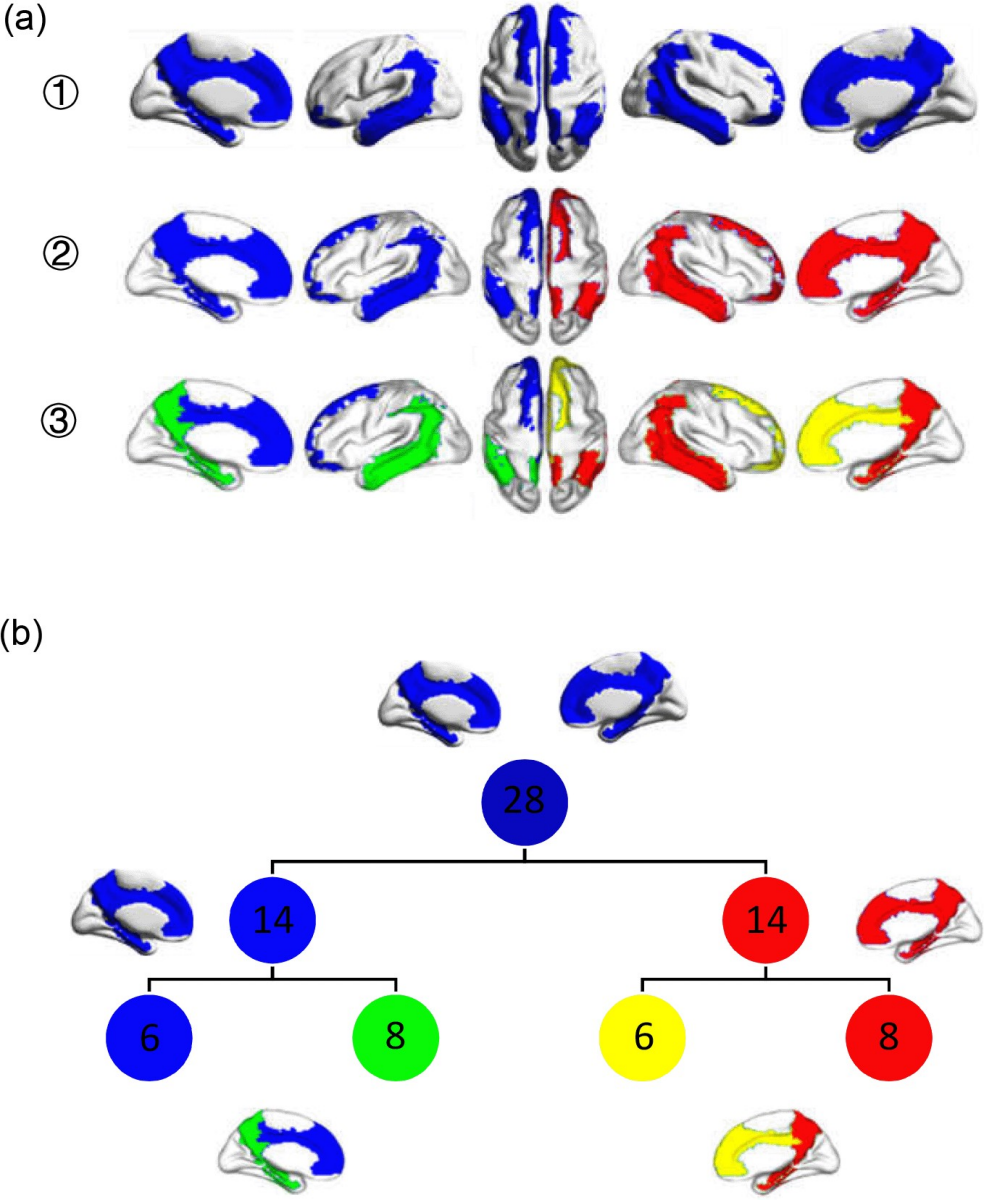
Hierarchical subnetworks of a DMN system. **(a)** The DMN system is divided into three levels. The 1^st^ level contains all the DMN regions. The 2^nd^ level consists of two subnetworks within each hemisphere of the DMN, i.e., the left DMN (lDMN) and the right DMN (rDMN). Each hemisphere of the DMN is divided into two subnetworks in the 3^rd^ level, i.e., the right anterior DMN (raDMN), the right posterior DMN (rpDMN), the left anterior DMN (laDMN) and the left posterior DMN (lpDMN). **(b)** The ROI number of each subnetwork in each DMN level. The 1^st^ level of the DMN includes 28 ROIs, and the rDMN and lDMN each contain 14 ROIs. At the highest level, the laDMN and raDMN each consist of 6 ROIs, and the number of ROIs in lpDMN and rpDMN are 8 respectively.

The asymmetries of the human brain, in terms of structure and function, have been well-documented and are involved in a variety of functions, such as language, motor, and visuospatial processing [32]. Studies focusing on hemisphere-related differences have revealed significant differences between the right and left hemispheres in the topological organization of functional networks in various brain regions, especially within the DMN [33, 34]. Recently, the intrinsic asymmetry activation patterns of the DMN were observed with an eigenmode-based hierarchical partition, which supports asymmetric functions in the DMN. According to the hierarchical partition shown in Fig 1, the DMN can be divided into three levels due to its limit regions. The 1^st^ level contains all DMN regions. The 2^nd^ level consists of two subnetworks within each hemisphere of the DMN, i.e., the left DMN (lDMN) and the right DMN (rDMN). Each hemisphere of the DMN is divided into two subnetworks in the 3^rd^ level, i.e., the right anterior DMN (raDMN), the right posterior DMN (rpDMN), the left anterior DMN (laDMN) and the left posterior DMN (lpDMN).

### Network analysis

The clustering coefficient and shortest path length from graph theory were used to characterize the capability of functional segregation and integration in brain functional networks [35]. The clustering coefficient of a network is defined as:

$$CC=\frac{1}{n}\sum_{i\in N}\frac{2t_{i}}{k_{i}(k_{i}-1)}$$ (2)

where *n* is the number of nodes, *N* is the set of all nodes in the network, *t*_*i*_ is the number of edges between the neighbors of node *i*, and *k*_*i*_ is the degree of node *i*, which represents that node’s number of neighbors. The clustering coefficient quantifies the number of connections that exist between the nearest neighbors of a node as a proportion of the maximum number of possible connections. Thus, a higher clustering coefficient implies a higher extent of segregation in a network. The shortest path length of a network is:

$$L=\frac{1}{n}\sum_{i\in N}\frac{\sum_{i\in N,j\neq i}d_{ij}}{n-1}$$ (3)

Here, *d*_*ij*_ is the shortest path length between nodes *i* and *j* of the network, which is an inverse of the connection weight between nodes *i* and *j* in a weighted correlation network. Note that *d*_*ij*_ = ∞ for all disconnected pairs *i* and *j*. Shorter paths imply a stronger potential for integration in a network.

We also adopted the participation coefficient to estimate the segregated and integrated network states. The participation coefficient expresses the level at which a node is coupled with other nodes across all modules and is described as:

$$P_{i,t}=1-\sum_{s=1}^{N_{M}}{\left(\frac{\kappa_{is,t}}{k_{i,t}}\right)}^{2}$$ (4)

where *κ*_*is*,*t*_ is the strength of the positive edge weights of node *i* to the nodes in module *s* at time *t*, *k*_*i*,*t*_ is the strength of the positive edge weights of node *i* to all the other nodes at time *t*, and *N*_*M*_ is the number of modules. A high value of *P*_*i*,*t*_ indicates that a node is connected to other nodes in most of the modules in a network, and low values of *P*_*i*,*t*_ indicate that a node is only connected to other nodes in a single or a small number of modules. A high mean *P*_*i*,*t*_ over nodes can be associated with the existence of highly integrative processes across the whole network. Here, all calculations in our network analyses are based on the Brain Connectivity Toolbox (BCT, https://www.nitrc.org/projects/bct/) in MATLAB.

## Results

### Functional segregation and integration in the hierarchical DMN

**Fig 2:**
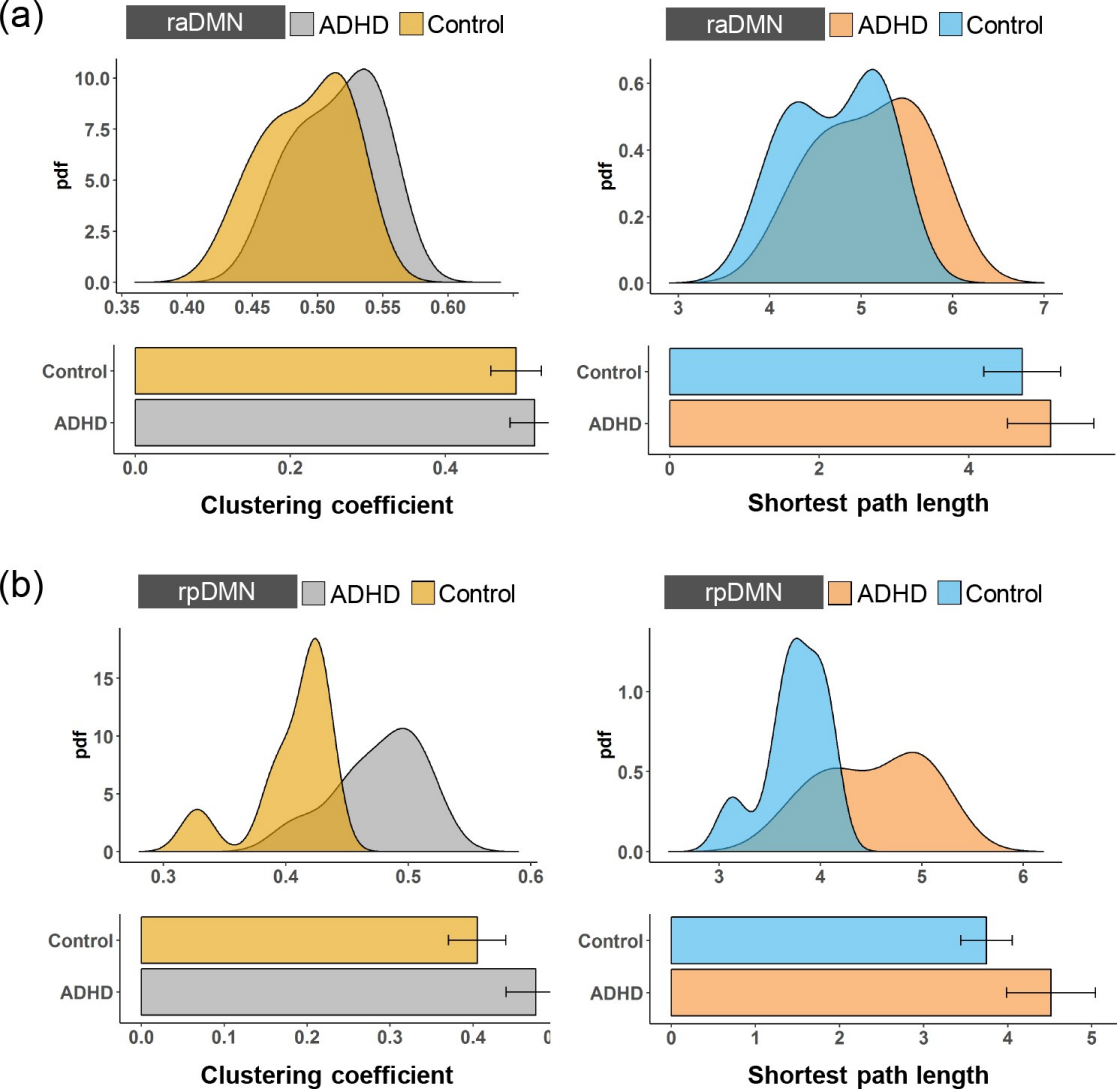
The probability density functions (pdfs) and boxplots of the clustering coefficient and the shortest path length for **(a)** the raDMN and **(b)** the rpDMN of the ADHD patients and the control subjects.

**Fig 3:**
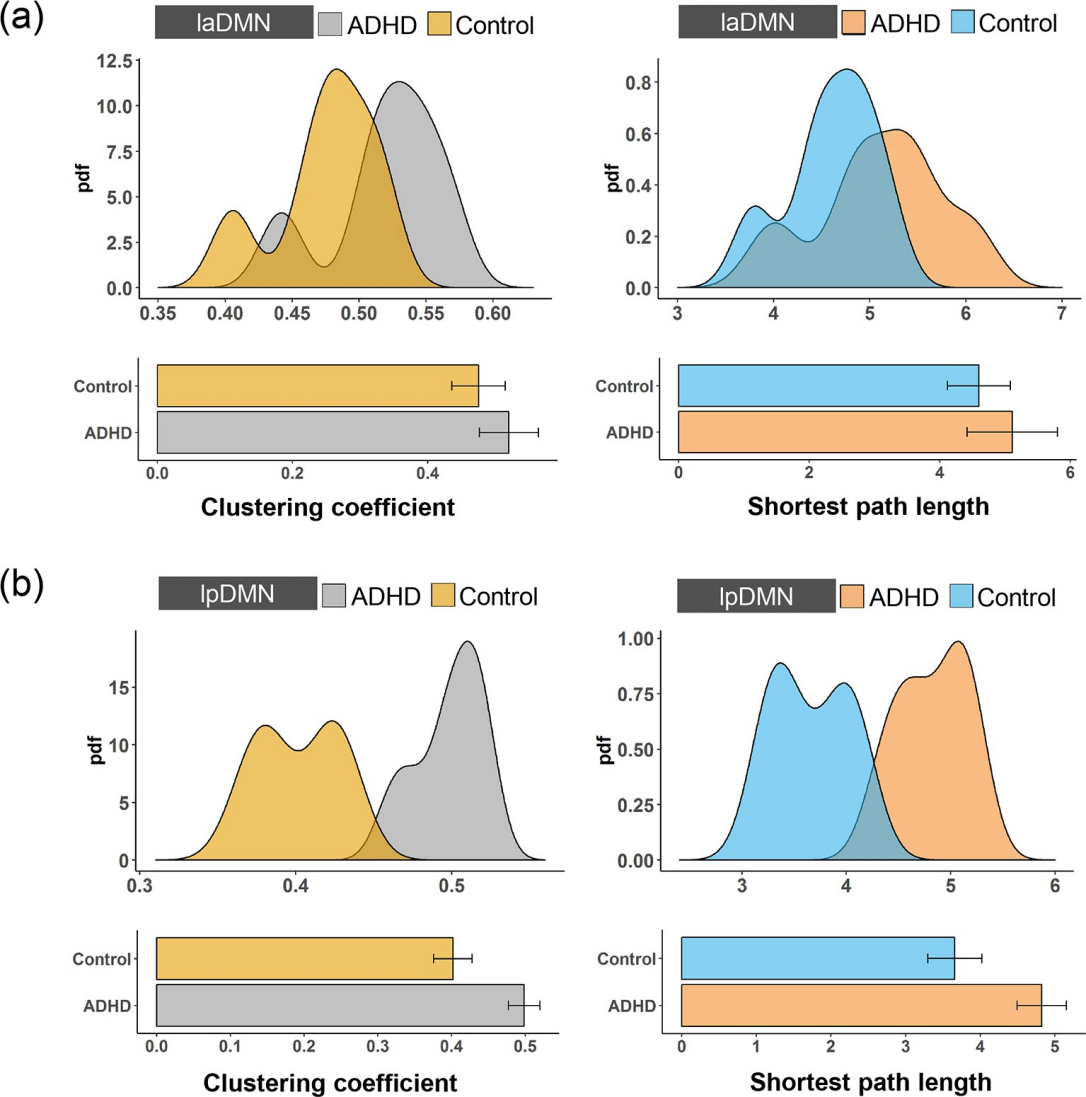
The pdfs and boxplots of the clustering coefficient and the shortest path length for **(a)** the laDMN and **(b)** the lpDMN of the ADHD patients and the control subjects.

The highest-level of the DMN contains four subnetworks, i.e., raDMN, rpDMN, laDMN and lpDMN (Fig 1). To investigate how ADHD affects the functional properties in the highest-level of the DMN, we first calculated the clustering coefficient and the shortest path length to measure the functional segregation and integration in each subnetwork [36]. The clustering coefficients for the four subnetworks in the ADHD patients were higher than those in the control subjects, as shown in Fig 2 and Fig 3. To further confirm any significant differences, we performed a two-sample t-test and found that the changes in the clustering coefficients in the lpDMN and rpDMN were statistically significant (*p < 0*.*05*; exact *p* values are listed in Table 1). However, this was not the case for the laDMN and raDMN (*p > 0*.*05*). We also found an increased shortest path length in the raDMN, rpDMN, laDMN and lpDMN in ADHD patients. By performing the statistical two-sample t-test, we confirmed that the changes in the shortest path length caused by ADHD were significant for the lpDMN and rpDMN (*p < 0*.*05*) but not for the laDMN and raDMN (*p > 0*.*05*).

**Table 1:** Exact *p* values of each two-sample t-test.

	Clustering coefficient	Shortest path length	Integration proportion	Frequency of transition	Functional connectivity
**DMN**	<0.001	0.046	0.735	0.048	-
**lDMN**	<0.001	<0.001	0.182	0.010	-
**rDMN**	<0.001	<0.001	0.041	0.332	-
**raDMN**	0.225	0.255	0.907	0.942	-
**rpDMN**	0.001	0.003	0.118	0.162	-
**laDMN**	0.092	0.166	0.216	0.023	-
**lpDMN**	<0.001	<0.001	0.188	0.098	-
**laDMN-lpDMN**	-	-	-	-	0.013
**raDMN-rpDMN**	-	-	-	-	0.116
**lDMN-rDMN**	-	-	-	-	<0.001

**Fig 4:**
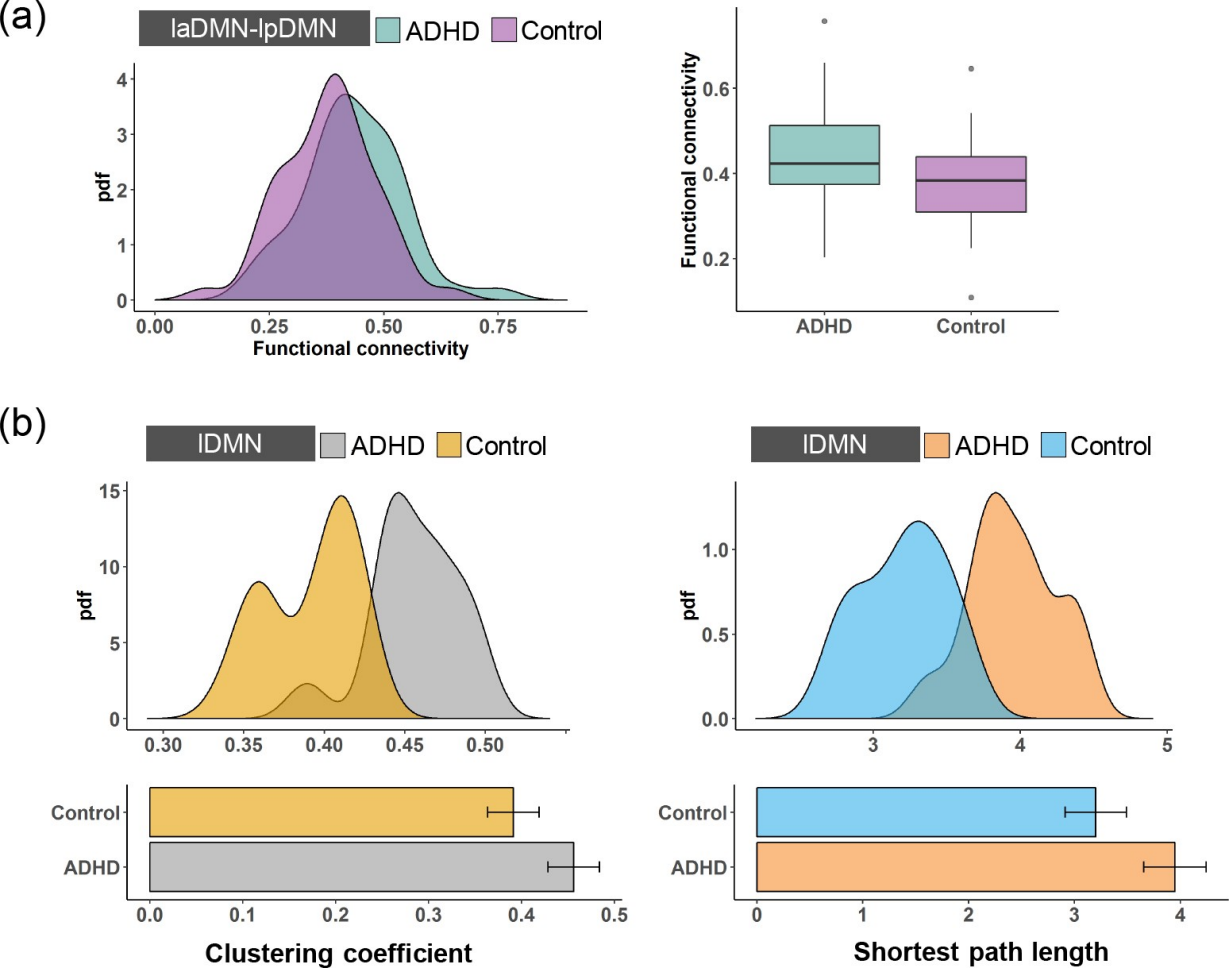
**(a)** The pdfs and boxplots of the functional connectivity of the laDMN-lpDMN for the ADHD patients and the control subjects. **(b)** The pdfs and boxplots of the clustering coefficient and the shortest path length for the lDMN of the ADHD patients and the control subjects.

We further investigated how the functional changes at the highest level were integrated to form abnormal segregation and integration at the 2^nd^ level, which contains the lDMN and the rDMN. Structurally, the lpDMN and laDMN are integrated into the lDMN, and thus, we first focused on the integration between these two modules. As shown in Fig 4A, the functional connectivity between the lpDMN and the laDMN in the ADHD patients was significantly larger than in the control subjects (two-sample t-test, *p < 0*.*05*), indicating a stronger functional integration between the lpDMN and the laDMN. Combining the decreased functional integration and increased segregation in the laDMN and lpDMN caused by ADHD, it is interesting to further investigate how the functional properties change in the lDMN. This result is shown in Fig 4B. We found that the clustering coefficient for the lDMN in the ADHD patients was higher than that in the control subjects, and this change is statistically significant (two-sample t-test, *p < 0*.*05*). Meanwhile, the shortest path length of the lDMN was significantly increased in the ADHD patients (two-sample t-test, *p < 0*.*05*), indicating decreased functional integration and increased functional segregation in the lDMN caused by ADHD.

**Fig 5:**
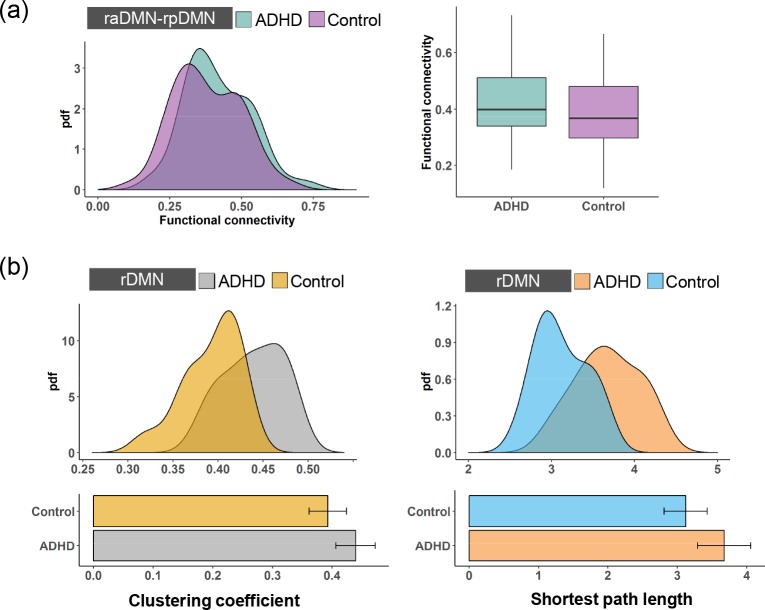
**(a)** The pdfs and boxplots of the functional connectivity of the raDMN-rpDMN for the ADHD patients and the control subjects. **(b)** The pdfs and boxplots of the clustering coefficient and the shortest path length for the rDMN of the ADHD patients and the control subjects.

**Fig 6:**
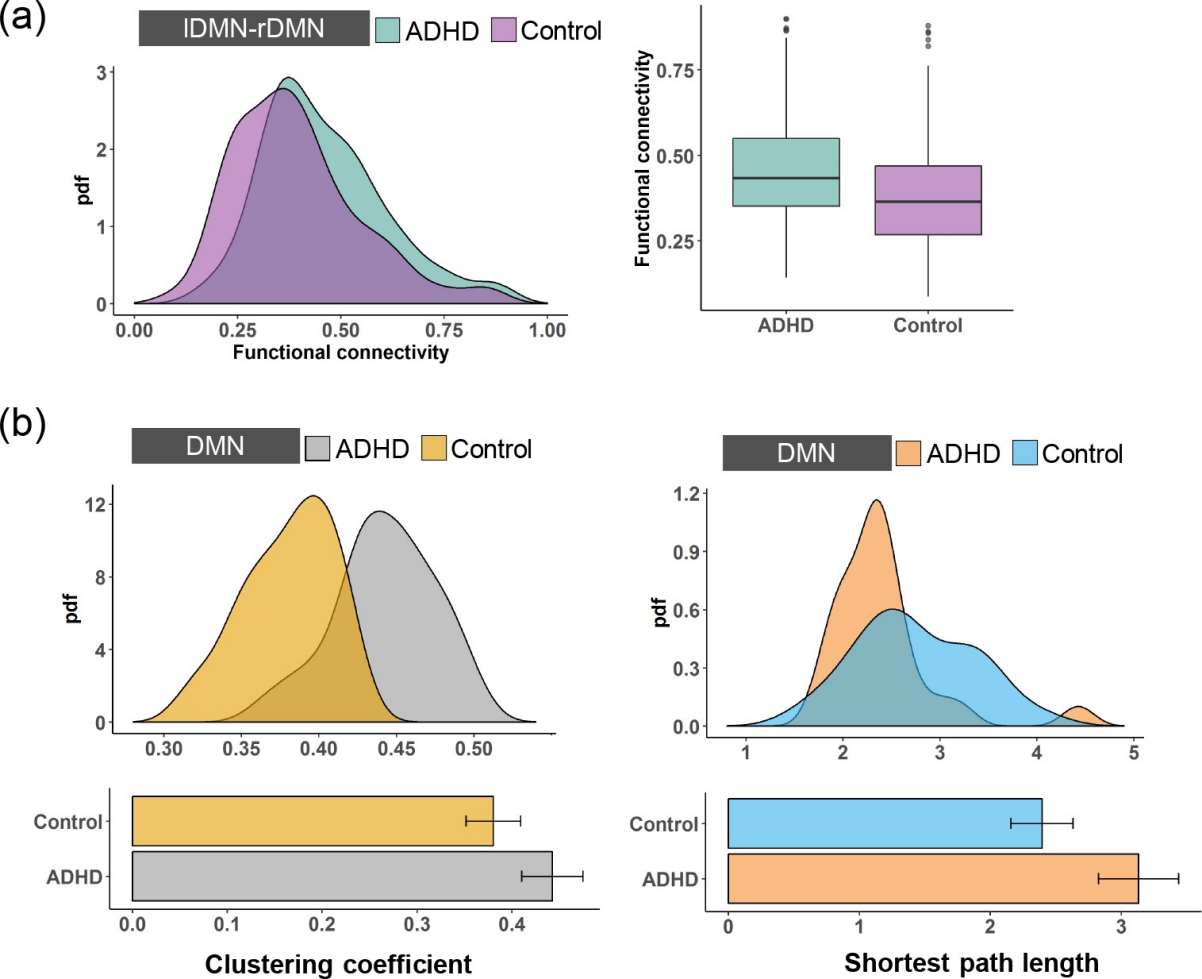
**(a)** The pdfs and boxplots of the functional connectivity of the lDMN-rDMN for the ADHD patients and the control subjects. **(b)** The pdfs and boxplots of the clustering coefficient and the shortest path length for the DMN of the ADHD patients and the control subjects.

Meanwhile, the rpDMN and raDMN are structurally integrated into the rDMN. As shown in Fig 5A, we also found that the functional connectivity between the raDMN and rpDMN in the ADHD patients was higher than that in the control subjects. However, this change was not statistically significant (two-sample t-test, *p > 0*.*05*), indicating that ADHD has an insignificant effect on the integration between the rpDMN and raDMN. We then calculated the clustering coefficient and the shortest path length for the rDMN. As shown in Fig 5B, the clustering coefficient and the shortest path length of rDMN in the ADHD patients were significantly larger than those in the control subjects (two-sample t-test, *p < 0*.*05* for both the clustering coefficient and the shortest path length). Thus, ADHD induces decreased functional integration and increased segregation in the rDMN by affecting the functional properties in the highest-level subnetworks. Apparently, the lDMN and rDMN can be integrated into the whole DMN. Fig 6A shows that the functional connectivity between the lDMN and rDMN is statistically improved in ADHD (two-sample t- test, *p < 0*.*05*), indicating stronger integration between the lDMN and rDMN. This is similar to the result for the lDMN. Furthermore, the clustering coefficient and the shortest path length for the DMN are significantly higher in the ADHD patients than in the control subjects (Fig 6B, two-sample t-test, *p < 0*.*05*), indicating decreased functional integration and increased segregation. Here, our results reveal that abnormalities in the DMN are intrinsically aroused by the changes in functional segregation and integration in the higher-level hierarchies.

### Dynamic transitions between functional segregation and integration in the hierarchical DMN

**Fig 7:**
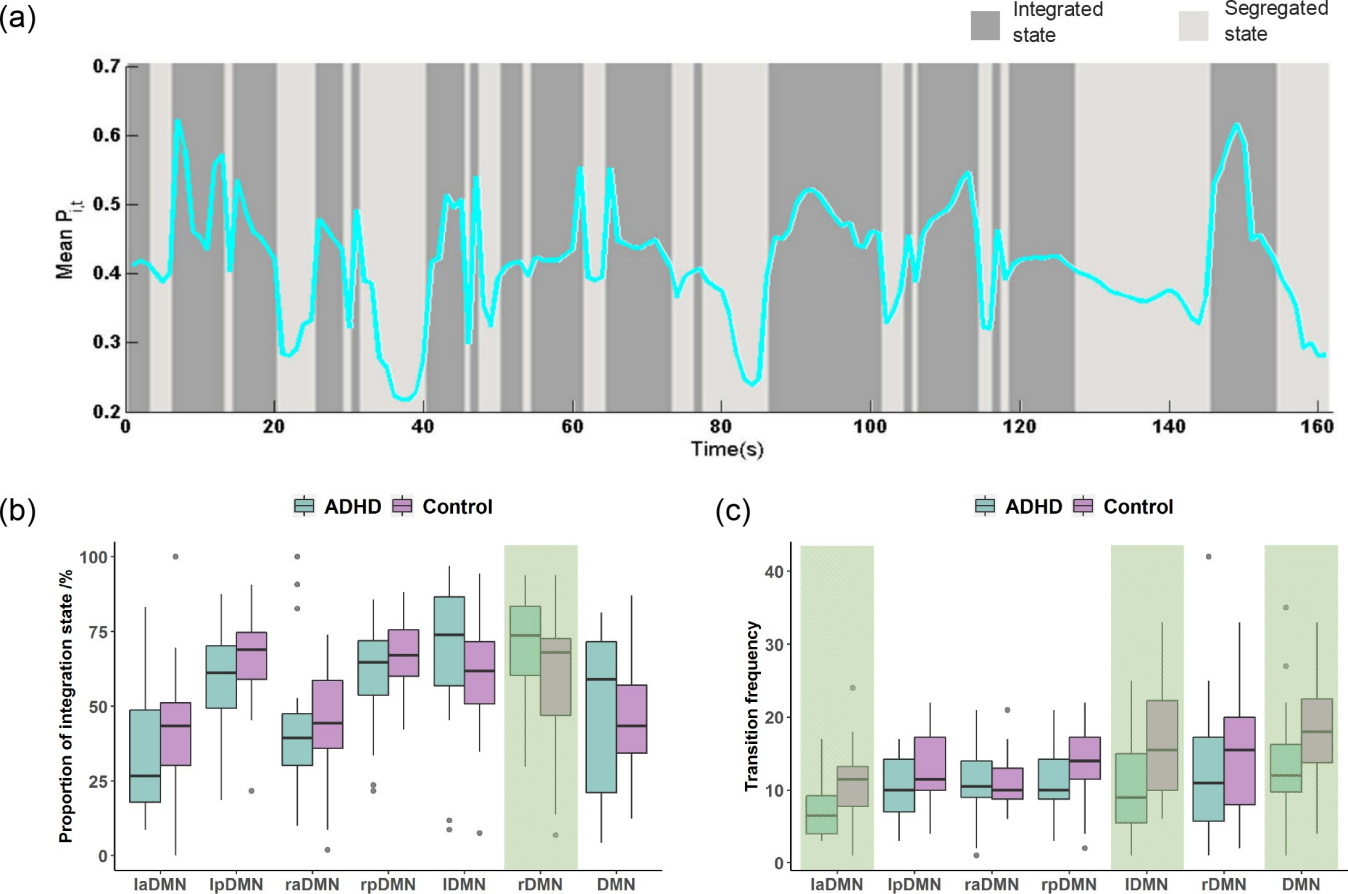
**(a)** The time series of the mean *P*_*i*,*t*_ and a sequence of state transitions in a representative subject. **(b)** Boxplots showing the proportion of the state of integration in a time series. **(c)** Boxplots showing the number of transitions between segregated and integrated states. The shaded portions of **(b)** and **(c)** represent the difference between the ADHD patients and the control subjects and is significant (two-sample t-test, *p < 0*.*05*).

Brain functional networks dynamically switch between segregated and integrated processing, and these flexible transitions consist of specific patterns that are dependent on brain state [37–40]. To investigate whether ADHD affects transitions between functional segregation and integration across the multiple levels the of DMN, we used the participation coefficient of dynamic functional networks to measure the flexible transitions in the brain. Computed from time-resolved functional networks, the states of network segregation and integration are estimated by classifying the participation coefficient in each time window using a *k*-means clustering algorithm (*k = 2*). The cluster with higher participation coefficients on average is regarded as the cluster of the integrated state. As shown in Fig 7A, the participation coefficient varies over the time course of transitions between functionally segregated and integrated states.

To investigate the dynamic integration ability, we calculated the proportion of the integration state over time for different subnetworks (Fig 7B). The mean values of the proportion of the integration state over time for the highest level networks, i.e., the laDMN, lpDMN, raDMN and rpDMN in the ADHD patients were lower than those in the control subjects. However, the two-sample t-test results indicate that the changes for all four subnetworks were not statistically significant (*p > 0*.*05*). We further investigated the proportion of the integration state over time for subnetworks in the 2^nd^ level. Interestingly, we found that the mean values of the proportion of the integration state over time for the lDMN and rDMN in the ADHD patients were higher than those in the control subjects, and the change in the rDMN was statistically significant (two-sample t-test, *p < 0*.*05*). Note that this is contrary to the results from the highest-level networks. At the 1^st^ level, the mean values of the proportion of the integration state over time for the DMN also improved under the influence of ADHD (*p > 0*.*05*).

Flexible transitions between functional segregation and integration are crucial for normal brain cognitive functions. To investigate the effect of ADHD on brain flexibility across multiple levels of the DMN, we calculated the frequency of the transition between functional segregation and integration in the time series for each subnetwork (Fig 7C). The four subnetworks in the highest level performed fewer transitions between functional segregation and integration states in the ADHD patients. In addition, the two-sample t-test showed that the change in transition frequency caused by ADHD is significant for the laDMN (two-sample t-test, *p < 0*.*05*). As integrated to lDMN and rDMN, the number of flexible transitions was also reduced in the ADHD patients, and this change was significant for lDMN (two-sample T-test, *p < 0*.*05*) but not for rDMN (*p > 0*.*05*). As mentioned before, the lDMN and rDMN are integrated into the whole DMN. Significantly fewer transitions between the functional segregation and integration states were performed in the DMN for the ADHD patients (*p < 0*.*05*).

## Discussion

In this study, we investigated functional segregation and integration features between ADHD patients and control subjects across hierarchical subnetworks of the DMN system. We found that abnormalities within the subnetworks of the DMN in ADHD are heterogeneous, and a dysfunctional DMN is essentially caused by changes in functional segregation and integration of its higher-level subnetworks. More interestingly, through the analysis of hierarchical dynamic transitions, we also found that the states of functional segregation and integration in the ADHD patients are more stable, which means that there is less adaptive regulation between the DMN subnetworks in the ADHD patients.

### ADHD brain network segregation and integration in the hierarchical DMN

We investigated the difference in the functional segregation and integration of the hierarchical DMN between the ADHD patients and the control subjects. We used a method to provide a hierarchical modular organization, which has a strong correspondence between the structure and functions of the DMN [33, 34]. In this hierarchical modular organization, the 1^st^ level contains all DMN regions, the 2^nd^ level includes the lDMN and rDMN, and the 3^rd^ level consists of the raDMN, rpDMN, laDMN and lpDMN.

The DMN is associated with attention-related brain cognitive functions. A dysfunctional DMN is often linked to attention deficits and mental diseases, such as depression and ADHD [18–20]. Our results show that the hierarchical DMN of ADHD exhibits different abnormal functional segregation and integration compared with control subjects. In the 3^rd^ level of the DMN, the clustering coefficient and the shortest path length of the lpDMN and rpDMN in the ADHD patients are significantly higher than in the control subjects, but these network metrics are not significant for the laDMN and raDMN. In the 2^nd^ level of the DMN (i.e., the lDMN and rDMN), the functional connectivity between the lpDMN and laDMN is significantly increased in ADHD patients, but it is not significantly different between the rpDMN and raDMN. More importantly, the clustering coefficient and shortest path length for both the lDMN and rDMN in the ADHD patients were statistically higher than those in the control subjects. It is further observed that the functional connectivity between the lDMN and rDMN is significantly increased in the ADHD patients, and the clustering coefficient and shortest path length for the DMN in the ADHD patients are statistically higher than those in the control subjects.

A higher clustering coefficient indicates that there are multiple communities in a subnetwork, which means that the functional segregation capability is increased in ADHD patients. Higher shortest path length means that the ability to process information is decreased in ADHD patients, and the functional integration capability is weakened. Many previous studies have indicated increased functional segregations and decreased functional integrations in the brain networks of ADHD patients [10, 13, 41]. Our results are consistent with previous studies. Furthermore, the changing of functional segregations and integration can also explain the increases in local brain efficiency and decreases in global efficiency found within ADHD [13].

Recently, studies have demonstrated the functional heterogeneity of the different brain regions in the DMN [42, 43]. Each region of the DMN was differentially activated by distinct cognitive processes and mental disorders [44]. Our study further confirmed that the functional segregation capability is hyperactivated and that the functional integration capability is weakened for the posterior DMN (i.e., the lpDMN and rpDMN) in the ADHD patients. However, for the anterior DMN subnetworks, the difference in the laDMN and raDMN between the ADHD patients and control subjects was not significant. Indeed, posterior cingulate cortex (PCC) dysfunction has been consistently reported in ADHD [26, 45]. As part of the lpDMN and rpDMN in the present hierarchical organization, it has been shown that the PCC exhibits the highest degree of connectivity in structure and might represent a “structural hub” of the DMN [46]. More importantly, the DMN shows the highest agreement of structural-function connectivity in the brain [47]. Thus, the PCC might also represent a “functional core” of the DMN and should be considered the possible locus of dysfunction in patients with ADHD. This may explain the heterogeneity of abnormalities of the anterior DMN and the posterior DMN in ADHD patients.

More interestingly, the lDMN and rDMN are regularly integrated by the highest-level subnetworks (i.e., the laDMN and lpDMN integrate to form the lDMN, and the raDMN and rpDMN integrate to form the rDMN), and both show functional segregation hyperactivity and a decrease in functional integration capability. These results may indicate that the functional change in the posterior DMN plays a key role in ADHD. The overall network characteristics of the DMN are determined by different subnetworks. These findings may enhance our understanding of the dysfunction in the DMN in ADHD patients.

Furthermore, the interaction of the subnetworks within the DMN also makes some important contributions to the construction of brain network segregation and integration. It should be noted that functional connectivity represents the capability of brain areas/networks to interact with information. The increased functional connectivity of the laDMN-lpDMN and the lDMN-rDMN may suggest an increase in local efficiency (increase in network segregation) of hyperactivity information processing in ADHD. Indeed, a decrease in global brain efficiency (decrease in network integration) and an increase in local brain efficiency have been confirmed in juvenile ADHD patients and adults [13, 48]. Abnormal brain functional network topology causes the classic “small-world” characteristic, which is crucial for normal brain cognitive function, and shifts the brain functional network toward a more regular network topology. This kind of deficit in the DMN may be part of the reasons for explaining ADHD clinical symptoms.

### ADHD brain network dynamic transitions between functional segregation and integration in the hierarchical DMN

The fluctuation between segregated and integrated processing of brain functional networks plays an important role in arousal and attention required for supporting effective cognitive processing [37, 38]. These fluctuations are not random but are highly structured. Aberrant fluctuation behavior of brain functional networks is linked to cognitive decline [39]. Here, the proportion of the integration state and the transition frequency between functional segregation and integration were used to measure fluctuation behavior of the DMN system in ADHD. For the 3^rd^ level subnetworks in the ADHD patients, the proportion of the integration state was lower than that in the control subjects. However, for subnetworks in the 2^nd^ level and 1^st^ level in the ADHD patients, the proportion is higher than that in the control subjects (Fig 7B). The results indicate that the 3^rd^ level subnetworks in the ADHD patients functionally segregate most of the time, whereas subnetworks in the 2^nd^ level and 1^st^ level show more functional integration. The potential explanation is that the higher functional connectivity between the 3^rd^ level subnetworks causes the phenomenon of increased instances of functional integration in the 2^nd^ level and 1^st^ level. In addition, the frequency of transitions between functional segregation and integration states in the ADHD patients was lower than that in the control subjects for both the 3^rd^ level subnetworks and the low-level subnetworks (the 2^nd^ level and 1^st^ level) (Fig 7C). The more stable state maintenance means less adaptive regulation between the DMN subnetworks in ADHD compared with the control subjects. More importantly, we found that only the laDMN, lDMN and DMN showed significantly fewer state transitions, which indicates that the characteristics of DMN are determined by its structurally nested subnetworks.

The notion that brain dynamics work on a critical point is growing in support [49]. At that particular point, the brain traverses around the repertoire of brain dynamics and guarantees a fast response even to weak external stimulations. In addition, fluctuations in network topology are considered to be associated with distinct patterns of behavior in brain cognitive functions [50]. The disruption of dynamics in integration and segregation is potentially linked to cognitive decline in ADHD patients.

### Limitations

There were a number of limitations in our study. First, our current study is limited by the small sample size. Although we have evaluated the power result, the sample size should be increased in future work. Second, the data we used here was acquired from resting state fMRI, and future studies should include data from task-state fMRI to study the cognitive competence of ADHD. Third, the present study focused on the hierarchical DMN. More work and investigations on the other hierarchical brain functional networks or the whole brain functional network will be included in the future.

## Conclusions

In this study, we investigated both segregation and integration brain network topological metrics between ADHD and healthy controls. We provided new insights to explain the brain cognitive dysfunction of ADHD from the view of hierarchical functional segregation and integration. We found that abnormalities between subnetworks in the DMN in ADHD patients are heterogeneous, and DMN dysfunction is essentially caused by changes in functional segregation and integration of its higher-level subnetworks. In addition, through the analysis of hierarchical dynamic transitions, we also found that the states of functional segregation and integration in ADHD patients are more stable, which means that there is less adaptive regulation between DMN subnetworks in ADHD patients. Our results can help us better understand the abnormal brain network mechanism of ADHD and serve as a clinical biomarker of ADHD.
